# RETRACTION: Meta‐Analysis of Minimally Invasive Arthroscopy with Sodium Hyaluronate for Wound Healing of Knee Osteoarthritis Treatment in the Elderly

**DOI:** 10.1111/iwj.70474

**Published:** 2025-04-02

## Abstract

**RETRACTION:** ZhangF. , ZhangJ. , and WangT. , “Meta‐Analysis of Minimally Invasive Arthroscopy with Sodium Hyaluronate for Wound Healing of Knee Osteoarthritis Treatment in the Elderly,” International Wound Journal 21, no. 4 (2024): e14512, 10.1111/iwj.14512.38069524 PMC10958090

The above article, published online on 09 December 2023, in Wiley Online Library (http://onlinelibrary.wiley.com/), has been retracted by agreement between the journal Editor in Chief, Professor Keith Harding; and John Wiley & Sons Ltd. Following an investigation by the publisher, all parties have concluded that this article was accepted solely on the basis of a compromised peer review process. The editors have therefore decided to retract the article. The authors did not respond to our notice regarding the retraction.

**RETRACTION:** F. Zhang , J. Zhang , and T. Wang , “Meta‐Analysis of Minimally Invasive Arthroscopy with Sodium Hyaluronate for Wound Healing of Knee Osteoarthritis Treatment in the Elderly,” International Wound Journal 21, no. 4 (2024): e14512, 10.1111/iwj.14512.38069524 PMC10958090

The above article, published online on 09 December 2023, in Wiley Online Library (http://onlinelibrary.wiley.com/), has been retracted by agreement between the journal Editor in Chief, Professor Keith Harding; and John Wiley & Sons Ltd. Following an investigation by the publisher, all parties have concluded that this article was accepted solely on the basis of a compromised peer review process. The editors have therefore decided to retract the article. The authors did not respond to our notice regarding the retraction.

